# A Case of Dissociative Fugue Following Acute Psychosocial Stress in a Previously High-Functioning Adult

**DOI:** 10.1192/bjo.2026.11886

**Published:** 2026-06-30

**Authors:** Gayathri Rangith

**Affiliations:** Cygnet Health Care, Oldbury, United Kingdom

## Abstract

**Aims:**

Dissociative fugue is a rare dissociative disorder characterised by sudden, reversible loss of autobiographical memory, identity disturbance, and purposeful wandering, often precipitated by acute psychological stress. Due to its infrequency and overlapping presentation with psychosis, delirium, and substance-induced amnesia, dissociative fugue is frequently misdiagnosed. We present a case of dissociative fugue in a previously high-functioning adult, highlighting diagnostic clarity, multidisciplinary management, and the critical role of family involvement in recovery.

**Methods:**

A man in his late thirties with no known psychiatric or medical history was brought to hospital by police after being found wandering outdoors inadequately dressed during winter. He was unable to identify himself, his personal history, or recognise family and friends, although he adopted an alternative name. He appeared emotionally neutral about his identity loss and demonstrated preserved ability to follow ward routines independently. Orientation to time was impaired, with reduced awareness of seasonality, while orientation to place was intact. There were no hallucinations, delusions, thought disorder, or features of delirium. Physical observations remained stable throughout admission.

Extensive investigations, including neuroimaging and blood tests (including infection and metabolic screening), were normal. Urine toxicology was negative, with no evidence of intoxication or withdrawal. Organic, psychotic, neurological, and substance-induced causes were excluded, and a working diagnosis of dissociative fugue was formally established. Psychosocial assessment revealed a sudden relationship breakdown preceding symptom onset alongside occupational stress. Psychological formulation conceptualised the presentation as trauma-related dissociation with identity fragmentation.

**Results:**

Management focused on psychological and systemic interventions rather than pharmacological treatment. Input included trauma-informed psychological therapy, occupational therapy with graded re-exposure, and structured family involvement. Identity reintegration was facilitated through in-person family visits, video calls, photographs, familiar language cues, and supported visits to the patient’s home. A marked emotional breakthrough occurred during the third week of admission, when the patient became tearful upon seeing his brother, coinciding with gradual restoration of autobiographical memory. Retrospective consent was obtained once capacity returned.

**Conclusion:**

This case demonstrates that dissociative fugue is not merely a theoretical diagnosis but a real and reversible clinical presentation in contemporary psychiatric practice. Preserved executive functioning, emotional neutrality towards identity loss, and absence of psychotic features were key diagnostic clues. Without early diagnostic clarity, the patient was at significant risk of misdiagnosis as dementia, psychosis, or substance-induced amnesia, potentially leading to unnecessary pharmacological treatment or prolonged institutionalisation. Coordinated multidisciplinary care, early collaboration with police and community services, and family involvement were central to recovery.

